# Supplementary figures and images for: Periplaneta americana (L.) Extract PAS840 Promotes Ischemic Stroke Recovery by Inhibiting Inflammasome Activation
Source: Biology (Basel). 2025 May 22;14(6):589. doi: 10.3390/biology14060589 (PMC12189569; doi:10.3390/biology14060589)

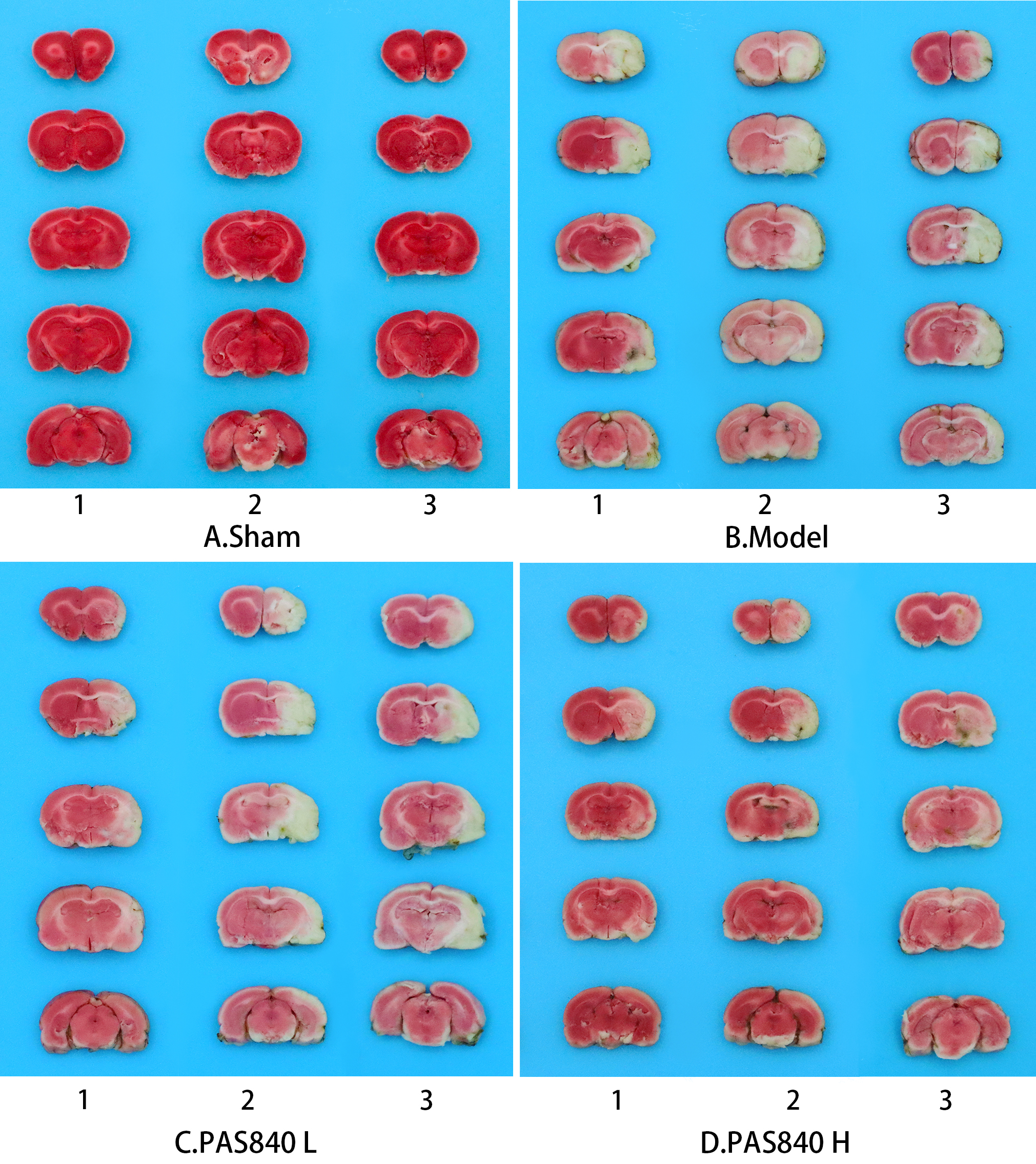

Supplement: Supplementary file 1 [file biology-14-00589-s001.zip › Figure S1 TTC staining.jpg]
